# Identification of potential nucleomodulins of *Mycoplasma bovis* by direct biotinylation and proximity-based biotinylation approaches

**DOI:** 10.3389/fmicb.2024.1421585

**Published:** 2024-07-09

**Authors:** Doukun Lu, Jiongxi Chen, Menghan Zhang, Yingjie Fu, Abdul Raheem, Yingyu Chen, Xi Chen, Changmin Hu, Jianguo Chen, Elise Schieck, Gang Zhao, Aizhen Guo

**Affiliations:** ^1^National Key Laboratory of Agricultural Microbiology, Huazhong Agricultural University, Wuhan, China; ^2^Hubei Hongshan Laboratory, Huazhong Agricultural University, Wuhan, China; ^3^College of Veterinary Medicine, Huazhong Agricultural University, Wuhan, China; ^4^Hubei International Scientific and Technological Cooperation Base of Veterinary Epidemiology, Huazhong Agricultural University, Wuhan, China; ^5^International Research Center for Animal Disease, Ministry of Science and Technology, Huazhong Agricultural University, Wuhan, China; ^6^International Livestock Research Institute, Nairobi, Kenya; ^7^Key Laboratory of Ministry of Education for Conservation and Utilization of Special Biological Resources in the Western China, School of Life Sciences, Ningxia University, Yinchuan, China

**Keywords:** *Mycoplasma bovis*, nucleomodulin, secreted proteins, direct biotinylation, proximity-based biotinylation

## Abstract

*Mycoplasma bovis* (*M. bovis*) is a significant bovine pathogen associated with various diseases, including bovine bronchopneumonia and mastitis resulting in substantial economic losses within the livestock industry. However, the development of effective control measures for *M. bovis* is hindered by a limited understanding of its virulence factors and pathogenesis. Nucleomodulins are newly identified secreted proteins of bacteria that internalize the host nuclei to regulate host cell gene expression and serve as critical virulence factors. Although recent reports have initiated exploration of mycoplasma nucleomodulins, the efficiency of conventional techniques for identification is very limited. Therefore, this study aimed to establish high-throughput methods to identify novel nucleomodulins of *M. bovis*. Using a direct biotinylation (DB) approach, a total of 289 proteins were identified including 66 high abundant proteins. In parallel, the use of proximity-based biotinylation (PBB), identified 28 proteins. Finally, seven nucleomodulins were verified to be nuclear by transfecting the bovine macrophage cell line BoMac with the plasmids encoding EGFP-fused proteins and observed with Opera Phenix, including the known nucleomodulin MbovP475 and six novel nucleomodulins. The novel nucleomodulins were four ribosomal proteins (MbovP599, MbovP678, MbovP710, and MbovP712), one transposase (MbovP790), and one conserved hypothetical protein (MbovP513). Among them, one unique nucleomodulin MbovP475 was identified with DB, two unique nucleomodulins (MbovP513 and MbovP710) with PBB, and four nucleomodulins by both. Overall, these findings established a foundation for further research on *M. bovis* nucleomodulin-host interactions for identification of new virulence factors.

## Introduction

Pathogenic mycoplasmal species, characterized by compact genomes (0.58–2.2 Mb) and the absence of cell walls, cause important diseases in both humans and animals. *Mycoplasma bovis* (*M. bovis*) can lead to mastitis, arthritis, keratoconjunctivitis, meningitis, otitis media, and genital tract diseases in cattle, as well as infertility and abortion (Dudek et al., 2020). However, *M. bovis* control is challenged by increasing antimicrobial resistance and the inefficacy of current vaccines (Gautier-Bouchardon et al., 2014; Perez-Casal, 2020). A comprehensive understanding of *M. bovis* pathogenesis is imperative for implementing evidence-based control measures.

The absence of cell walls in *Mycoplasma* facilitates the direct interaction of membrane-associated proteins with host cells, thereby acting as virulence factors. Consequently, previous investigations into virulence factors have predominantly focused on membrane surface proteins, such as *M. bovis* adhesins P27 (Chen et al., 2018), VpmaX (Zou et al., 2013), and Mbov_0503 (Zhu et al., 2020), as well as membrane-associated nucleases such as MBOV_RS02825 (Zhang et al., 2016), MBOVPG45_0215 (Sharma et al., 2015), and MGA_0676 (Xu et al., 2015). Secreted proteins play a crucial role in mediating communication between hosts and pathogens, serving as integral components that modulate both immunity and pathogenicity. Although Mycoplasma has been demonstrated to produce and release various pathogenic molecules, including polypeptides, exopolysaccharides, and extracellular vesicles (Gaurivaud and Tardy, 2022), research on the functions of Mycoplasma-secreted proteins remains relatively limited. Examples include the secreted serine protease S41 of *M. capricolum* (Ganter et al., 2019), the moonlight cytoadhesin EF-tu of *M. hyopneumoniae*, and the inflammatory/apoptosis inducers MbovP145 and MbovP280 of *M. bovis* (Lu et al., 2021; Zhao et al., 2021).

Bacterial pathogens use diverse effector proteins to reprogram host cells, often targeting the nucleus to modulate gene expression without altering the genetic sequence. These effector proteins are known as nucleomodulins (Hanford et al., 2021). The first nucleomodulins were found in plant pathogens like Agrobacterium and Xanthomonas, where they acted as transcription factors or integrated into host DNA to induce tumor formation (Hanford et al., 2021). Recently, many nucleomodulins have been discovered in mammalian pathogens. These bacterial proteins target various components of host cell control mechanisms, including chromatin dynamics, histone modification, DNA methylation, RNA splicing, DNA replication, cell cycle regulation, and cell signaling pathways. They induce short- or long-term epigenetic changes in host cells (Bierne and Cossart, 2012). For instance, nucleomodulin AnkA from *Anaplasma phagocytophilum* exhibits a binding affinity towards sequences of A, T, and C nucleotides within chromatin, leading to alterations in chromatin structure, facilitation of histone H3 deacetylation, and subsequent gene silencing in the host cell response (Rennoll-Bankert et al., 2015; Dumler et al., 2016; Escoll et al., 2016). Similarly, nucleomodulin Ank/p200 of *Ehrlichia chaffeensis* (*E. chaffeensis*) binds to chromatin via a specific interaction with AT-rich Alu-sx motifs within host genes, leading to significant changes in gene expression on a large scale (Zhu et al., 2009; McBride and Walker, 2011). Nucleomodulin BaSET derived from *Bacillus* targets eight lysine residues on histone H1, resulting in transcriptional repression (Mujtaba et al., 2013; Alvarez-Venegas, 2014; Mehta and Jeffrey, 2015). Nucleomodulin BtSET from *Burkholderi* targets H3K4 and nucleolar rDNA and demonstrates methyltransferase activity (Escoll et al., 2016). Likewise, nucleomodulin RomA from *Legionella pneumoniae* interacts with histone H3K14, inducing methylation that subsequently inhibits the expression of genes related to innate immunity and facilitates intracellular bacterial proliferation (Rolando et al., 2013). Similarly, the nucleomodulin NUE from *Chlamydia trachomatis* induces methylation of histones H2B, H3, and H4, as well as self-methylation (Pennini et al., 2010). Nucleomodulin Ptpa from *Mycobacterium tuberculosis* (*M. tb*) can translocate into the nuclei of U937 macrophages and bind various promoter regions within the host genome, including GADD45A (Wang et al., 2017). This interaction results in the suppression of immune responses. Another *M. tb* nucleomodulin, Rv2966c, functions as a methyltransferase, predominantly methylating cytosines in a non-CpG context (Sharma et al., 2015).

Moreover, certain nucleomodulins possess the ability to modulate signaling pathways. For instance, the nucleomodulin BopN from *Bordetella pertussis* has been implicated in binding or promoting export of p65 from the nucleus, thereby manipulating the host NF-κB pathway and facilitating nuclear translocation of p50 (Nagamatsu et al., 2009). Similarly, nucleomodulin Ank1 from *Orientia* engages with the CULLIN-1 and SKP1 components of the SCF-E3 ubiquitin ligase complex, potentially facilitating the binding or export of p65 from the nucleus to modulate host NF-κB signaling pathway (Min et al., 2014; Evans et al., 2018). The nucleomodulin Cbu0781 (AnkG) from *Coxiella burnetii* targets nuclear proteins, leading to alterations in host cell processes and delayed apoptosis (Lifshitz et al., 2014; Bisle et al., 2016). In addition to this, some studies have demonstrated that bacterial nucleomodulins could manipulate directly host cell signaling regulatory proteins. For instance, ligase activity of type I secreted tandem repeat protein (TRP120) of *E. chaffeensis* promotes ehrlichial infection by degrading F-box and WD repeat domain-containing 7 (FBW7), and maintaining the stability of Notch and other oncoproteins involved in cell survival and apoptosis (Wang et al., 2020). Further, TRP120 interacts with another regulatory protein, i.e., polycomb group ring finger protein 5 (PCGF5) and other PRC1 components. During infection, PCGF5 and other isoforms relocate from the nucleus to the ehrlichial vacuole, leading to their degradation. This disrupts PRC1 function and alters Hox gene transcription, enhancing *E. chaffeensis* infection (Mitra et al., 2018).

In the context of *mycoplasmas*, several types of nucleomodulins have been identified thus far. One is the DNA methyltransferase of *M. hyorhinis*, which targets specific DNA recognition sites to promote tumor progression (Chernov et al., 2015). The other type is the transcription regulator MbovP475 of *M. bovis*, which targets host CRYAB and MCF2L2 gene promoters to inhibit macrophage proliferation and promote intracellular survival (Zhao et al., 2022). In addition, *M. fermentans* DnaK, a chaperone protein belonging to the HSP70 family, can be taken-up by uninfected cells, and upon infection, localizes in different cellular compartments including the nucleus, where it interacts with poly (ADP-ribose) polymerase 1 (PARP1) and p53, thereby reducing DNA repair activities and anti-cancer responses (Zella et al., 2018; Benedetti et al., 2020). Nucleomodulins as novel virulence factors, play a pivotal role in the pathogenesis of *M. bovis*. To identify the potential nucleomodulins of *M. bovis* with high-throughput approaches, we designed two experimental approaches to explore nucleomodulins localized in host cell nuclei. We employed the direct biotinylation (DB) approach to enrich nucleomodulins from the cells treated with *M. bovis* proteins labeled with biotin *in vitro*. In addition, we used a proximity-based biotinylation (PBB) approach to enrich nucleomodulins from cells infected with *M. bovis*. The discovery of novel nucleomodulins through these approaches will contribute to more comprehensive understanding of the host-*M. bovis* interaction.

## Materials and methods

### Growth of cells and bacterial strains

The bovine macrophage cell line (BoMac) used in this research was generously donated by Judith R. Stabel from the Johne’s Disease Research Project (Stabel and Stabel, 1995). For cultivation, BoMac cells were cultured in RPMI 1640 (Hyclone, USA) supplemented with 10% heat-inactivated fetal calf serum (FBS) (Gibco, USA). The *M. bovis* HB0801 strain (GenBank accession no. NC_018077.1) was isolated from the lungs of diseased cattle in Hubei province (China) in 2008 (Qi et al., 2012) and grown in pleuropneumonia-like organism (PPLO) media (BD Company, MD, USA) as previously described (Han et al., 2015).

### Proteins of *Mycoplasma bovis* biotinylation *in vitro*

For the DB approach, *M. bovis* was cultured in 10 mL PPLO medium for 36 h and precipitated by centrifugation at 12,000 × *g* for 5 min. The pellet of *M. bovis* was suspended in 2 mL of phosphate-buffered saline (PBS) with 1× Halt Protease Inhibitor Cocktail (100×) (Thermo Fisher Scientific, Rockford, USA) and lysed by sonication at 200 W on ice for 15 min for a total of 60 cycles. Each sonication cycle was as follows: sonication 5 s, resting period 10 s. Whole cell lysate proteins of *M. bovis* were isolated in the supernatant after centrifugation at 12,000 × *g* for 10 min. Proteins concentration was measured with a BCA kit (Beyotime, China). Proteins were then labeled with biotin using the EZ-Link™ Sulfo-NHS-LC-Biotinylation Kit (Thermo Scientific, Rockford, USA). Biotin is a small naturally occurring vitamin that binds with high affinity to avidin and streptavidin proteins. Because of its size, biotin can be conjugated to many proteins without altering their biological activities. N-Hydroxysuccinimide (NHS) ester-activated biotins are the most popular type of biotinylation reagent. NHS esters react efficiently with primary amino groups (-NH2) in pH 7–9 buffers to form stable amide bonds. Because proteins generally contain multiple lysine (K) residues in addition to the N-terminus of each polypeptide, they have multiple primary amines available as targets for labeling with NHS-activated reagents. Generally, 10 mg whole cell proteins in 0.5 mL PBS mixed with 135 μL 10 mM Sulfo-NHS-LC-Biotin solution was used. The reaction was incubated on ice for 2 h and excess biotin reagent was removed using a desalting column.

BoMac cells at 80% confluency in a 10 cm dish were treated with 10 mg biotin- labeled proteins for 24 h. Cytoplasmic and nuclear proteins of BoMac cells were extracted using a Minute™ Cytoplasmic and Nuclear Fraction Kit (Invent, Beijing, China) according to the manufacturer’s instructions. Western blotting was used to assess contamination the nuclear proteins with cytoplasmic proteins. Proteins were resolved by SDS-PAGE and transferred to a PVDF membrane. Immunodetection was achieved with antibodies against α-tubulin as the internal reference for cytoplasmic proteins (Abcam, Cambridge, UK) or PARP (Abcam, Cambridge, UK) for nuclear proteins. Proteins were visualized with a WesternBright™ ECL western blotting detection kit (Advansta, San Jose, CA, USA). Nuclear proteins were incubated with 100 μL Dynabeads™ M-280 Streptavidin (Invitrogen, Carlsbad, CA, USA) for 2 h. After five washes with PBS, proteins were subjected to LC–MS/MS analysis (Applied Protein Technology Co. Ltd., Shanghai, China) for sequencing.

### BoMac cell infection model

To validate the *M. bovis* infection model, BoMac cells at a concentration of 1 × 10^5^ were pre-seeded on microscope coverslips or directly seeded in 12-well plates and incubated overnight at 37°C. The cells were infected with *M. bovis* at an MOI of 1,000 for 24 h. For indirect immunofluorescence assay (IFA), the BoMac cells on microscope coverlips were fixed with 4% paraformaldehyde for 10 min at room temperature. The fixed cells were blocked in 5% bovine serum albumin for 1 h followed by incubation with mouse anti-MbovP579 primary monoclonal antibodies overnight. After washing with PBS, the cells were treated with the secondary antibodies DyLight 594, goat anti-mouse IgG (Abbkine Scientific Co., Ltd., Wuhan, China) for 1 h at room temperature. Cell nuclei were counterstained with DAPI (Beyotime, Shanghai, China). Fluorescent images were acquired with confocal laser scanning microscope (Olympus FV1000, Tokyo, Japan). For western blotting, the proteins from infected BoMac cells were lysed using RIPA lysis buffer with 1 × Halt Protease Inhibitor Cocktail (100×) (Thermo Fisher Scientific, Rockford, USA). Immunodetection was achieved with antibodies against β-actin as the internal reference (Proteintech, Shanghai, China) or MbovP579 (prepared by our laboratory) by the method described above.

### Biotin-phenol labeling of live cells

For the PBB approach, BoMac cells in a 10-cm dish, as well as those pre-seeded on microscope coverslips in a 12-well plate, were transfected with the pEXP (V5_H2B_APEX2) plasmid (Addgene, Watertown, MA, USA; #107597) to transiently express nuclear APEX2-H2B with the V5 tag, followed by infection with *M. bovis* (MOI = 1,000) for 24 h. BoMac cells infected with *M. bovis* served as a control. Biotin-phenol (Sigma-Aldrich, St. Louis, MO, USA) was added into the dish at a final concentration of 0.5 mM and cells were incubated at 37°C for 30 min. H_2_O_2_ (1 mM) was added to the dish to initiate biotinylation. After 1 min, biotinylation was terminated by removing the media, followed by five consecutive washes with a quencher solution (10 mM sodium azide, 10 mM sodium ascorbate, and 5 mM Trolox in PBS). APEX is able to oxidize phenol derivatives (such as phenol-biotin) to phenoxyl radicals that covalently react with electron-rich amino acids such as Tyr and Trp in proteins (Rhee et al., 2013). Because the biotin-phenoxy radical produced by the APEX2 labeling process is short-lived (< 1 ms) and can produce a smaller labeling radius (< 20 nm), APEX2 labeling can provide higher space and time specificity (Lam et al., 2015). H2B is a histone that localizes within the host cell nucleus, capable of transporting APEX2 into the nucleus of BoMac cells and biotinylating proteins of *M. bovis* as well as those within the BoMac cell nucleus within a range of 20 nm.

To assess the feasibility of the PBB approach, the BoMac cells processed as described above, pre-seeded on microscope coverslips, were fixed with 4% paraformaldehyde for IFA. The cells were treated with the antibody mouse anti-V5 tag primary monoclonal antibodies, then incubated with the secondary antibodies Alexa Fluor 488-labeled goat anti-mouse IgG (Life Technologies, Thermo Fisher Scientific, Carlsbad, CA, USA) and streptavidin conjugate Alexa Fluor 568 (Invitrogen). The location of APEX2-H2B and proteins labelled with biotin was observed using a confocal laser scanning microscope (Olympus FV1000, Tokyo, Japan).

After verifying the nuclear location of proteins labelled with biotin, we used the PBB approach to process BoMac cells. The cells were scraped and collected by centrifugation for 10 min at 3000 × *g* at 4°C. Cell pellets were lysed using RIPA lysis buffer with 1× Halt Protease Inhibitor Cocktail (100×) (Thermo Fisher Scientific, Rockford, USA), 1 mM PMSF, and quenchers (10 mM sodium azide, 10 mM sodium ascorbate, and 5 mM Trolox). Dynabeads M-280 Streptavidin (Invitrogen, USA), washed three times with RIPA buffer, were incubated with corresponding whole cell proteins in 1.8 mL RIPA buffer overnight at 4°C with gentle rotation. A magnetic separator (Thermo Fisher Scientific, USA) was used to facilitate the separation of beads from the buffer solution. The beads underwent a series of washes, including two with 0.2% SDS, one with buffer 1 (20 mM Tris pH 7.5 and 2% SDS), two with buffer 2 (0.1% DOC, 1% Triton X-100, 500 mM NaCl, 1 mM EDTA, and 50 mM HEPES, pH 7.5), one with buffer 3 (250 mM LiCl, 0.5% NP-40, 0.5% DOC, 1 mM EDTA, and 10 mM Tris pH 8.1), two with buffer 4 (50 mM Tris, pH 7.4 and 50 mM NaCl), and two with PBS. The proximity-based biotinylated proteins were then eluted from the beads by boiling in 80 μL of SDT buffer (4% (w/v) SDS, 100 mM Tris–HCl, 1 mM DTT, pH 7.6). Following elution, a quarter of the proteins were analyzed via silver staining after SDS-PAGE. The remaining samples were sequenced by LC–MS/MS for protein identification (Applied Protein Technology Co. Ltd., Shanghai, China).

### Mass spectrometry analysis

The samples enriched by the above two methods were diluted to a final volume of 100 μL using 8 M urea (UA). Then, 2 μL of 1 M dithiothreitol (DTT) was added, and the solution was incubated with agitation at room temperature for 2 h to reduce disulfide bonds. Subsequently, 40 μL of 100 mM iodoacetamide (IAA) was added, and the sample was incubated in the dark at room temperature for 30 min. Then, 600 μL of 25 mM ammonium bicarbonate (NH_4_HCO_3_) buffer was added, and the mixture was agitated and centrifuged to remove insoluble components. Finally, 3 μg of trypsin was added, and the digestion was performed with agitation at 37°C for 16–18 h to enzymatically cleave the proteins into peptides. The C18 desalting column was used for desalination treatment. After lyophilization, the peptides were reconstituted in 40 μL of 0.1% formic acid solution.

LC–MS/MS data acquisition was carried out on a Q Exactive HF mass spectrometer (Thermo Fisher Scientific, USA) coupled with an UltiMate 3,000 RSLCnano system (Thermo Fisher Scientific, USA). Peptides were first loaded onto a C18 trap column (75 μm × 2 cm, 3 μm particle size, 100 Å pore size, Thermo Scientific) and then separated in a C18 analytical column (75 μm × 25 cm, 1.9 μm particle size, 100 Å pore size, Thermo Fisher Scientific, USA). Mobile phase A (0.1% formic acid/3% DMSO/97% H_2_O) and mobile phase B (0.1% formic acid/3% DMSO/97% acetonitrile) were used to establish the separation gradient at a flow rate of 300 nL/min. For analysis in data-dependent acquisition (DDA) mode, each scan cycle consisted of one full-scan mass spectrum (resolution = 60 K at m/z 200, AGC = 3e6, max IT = 30 ms, scan range = 350–1800 m/z) followed by 20 MS/MS events (resolution = 15 K at 200 m/z, AGC = 1e5, max IT = 50 ms). Dynamic exclusion was enabled and set to 30s.

MS raw data were analyzed with MaxQuant (V1.6.6.0) using the Andromeda database search algorithm. The *M. bovis* HB0801 proteome database contained 873 protein sequences download from the NCBI database. Search results were filtered with a 1% false discovery rate (FDR) at both protein and peptide levels. Proteins denoted as reverse, or only identified by sites were removed, and the remaining proteins were used for further analysis.

### Cloning recombinant plasmids

Mbov_0116, Mbov_0211, Mbov_0478, Mbov_0599, Mbov_0678, Mbov_0712, Mbov_0049, Mbov_0157, Mbov_0160, Mbov_0255, Mbov_0353, Mbov_0401, Mbov_0710, and Mbov_0790 were cloned from the *M. bovis* genome with overlapping PCR. Primers used to amplify these fragments are listed in Supplementary Table S1. Site-directed mutagenesis was used to alter the *M. bovis* TGA codon into TGG to ensure that *M. bovis* tryptophan was encoded in mammalian cells. The recombinant plasmids pEGFP-116, pEGFP-211, pEGFP-478, pEGFP-599, pEGFP-678, pEGFP-712, pEGFP-049, pEGFP-157, pEGFP-160, pEGFP-255, pEGFP-353, pEGFP-401, pEGFP-710, and pEGFP-790 were constructed by inserting the above fragments into the pEGFP-C1 vector. Meanwhile, the pEGFP-513, pEGFP-579, pEGFP-582, pEGFP-663, pEGFP-699, pEGFP-791, and pEGFP-798 eukaryotic expression plasmids encoding the *egfp* gene fused with mycoplasmal genes were constructed with a similar strategy by Beijing Tsingke Biotech Co., Ltd. (Whuhan, China). The eukaryotic expression plasmids pEGFP-280 and pEGFP-475 were previously prepared by our laboratory (Zhao et al., 2022). All constructed plasmids were verified by DNA sequencing (Beijing Tsingke Biotech, Wuhan, China).

### Immunofluorescence observation of transfected BoMac cells

BoMac cells at a concentration of 1 × 10^5^ were seeded in each well of 12-well plates overnight. The cells were transfected with 0.8 μg pEGFP-513, pEGFP-582, pEGFP-663, pEGFP-699, pEGFP-791, pEGFP-798, pEGFP-049, pEGFP-116, pEGFP-157, pEGFP-160, pEGFP-211, pEGFP-255, pEGFP-353, pEGFP-401, pEGFP-478, pEGFP-579, pEGFP-599, pEGFP-678, pEGFP-710, pEGFP-712, pEGFP-790, pEGFP-280, or pEGFP-475 with 1.5 μL Lipofectamine 2000 (Invitrogen, Carlsbad, CA, USA). At 24 h, the cells were fixed with 4% paraformaldehyde for 10 min at room temperature. After washing with PBS, nuclei were stained with DAPI (Beyotime, Shanghai, China). Fluorescent images were acquired with the Opera Phenix (PerkinElmer life and Analytical Sciences Ltd., Waltham, MA, USA).

### *In silico* comparative analyses of the functions of enriched proteins

*In silico* functional analysis of the potential nucleomodulin proteome was performed as previously described with minor modifications (Dawood et al., 2023). Classically secreted proteins that carry signal sequences were predicted by the SignalP 6.0 server.^1^ No signal peptide-triggered protein secretion was searched using the SecretomeP 2.0 server.^2^ If proteins were predicted to be neither classical nor non-classical secretory proteins, they were classified as “Undefined.” In addition, COG functional annotation for the proteins identified was acquired using the EggNOG database version 5.0.^3^ The Retrieve/ID Mapping tool from UniProt^4^ was used to assign subcellular locations to identified host proteins.

## Results

### Identification of nucleomodulins by the DB approach

**Figure 1:**
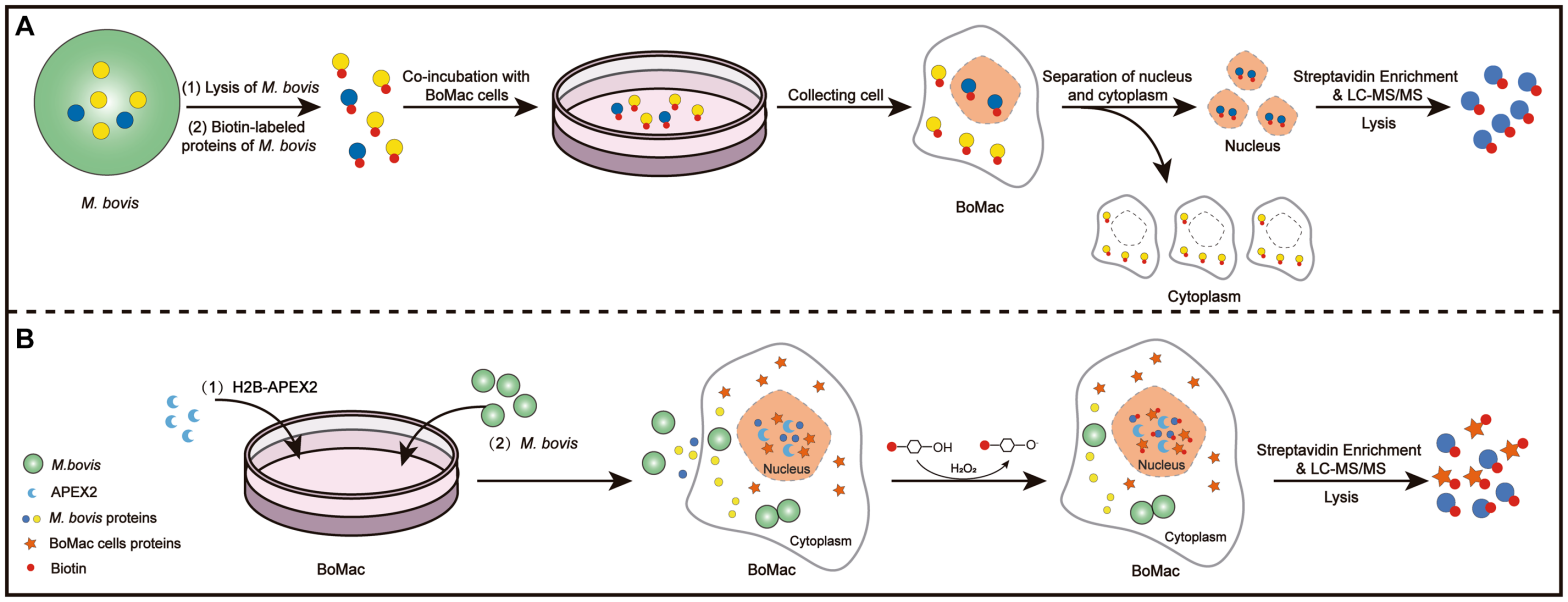
Schematic illustration of the DB **(A)** and PBB **(B)** approaches.

**Figure 2:**
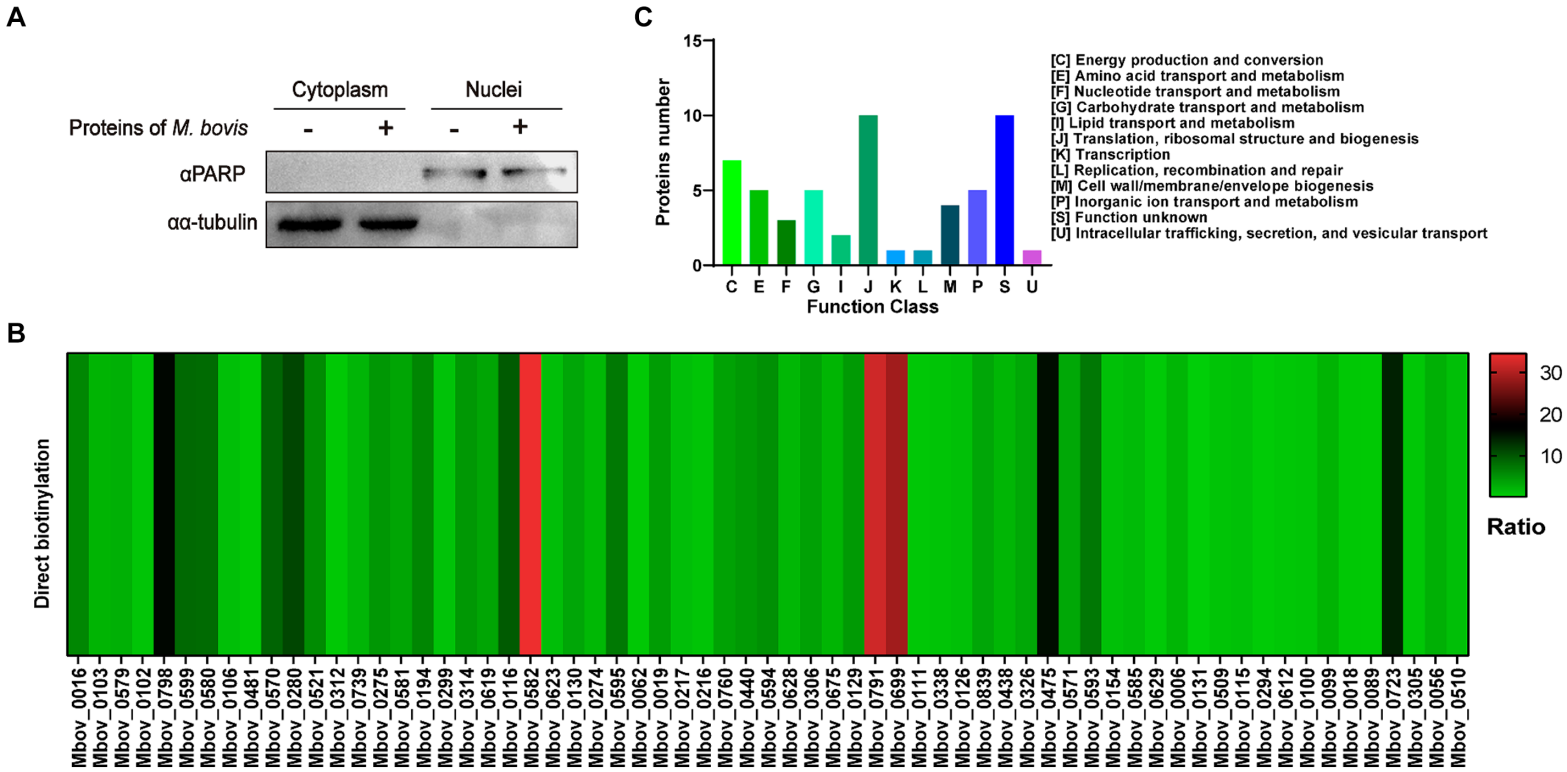
Identification of potential nucleomodulins by the DB approach. **(A)** After co-incubation with biotin-labeled *M. bovis* protein, the nuclei and cytoplasm were isolated and detected. **(B)** The ratio between the IBAQ of 66 proteins in DB approach and these proteins in the total protein of *M. bovis*. **(C)** The COG functional distribution of potential *M. bovis* nucleomodulins by DB approach.

As shown in Figure 1A, we collected samples of BoMac cells incubated with *M. bovis* proteins labeled with biotin. The nuclear proteins were extracted and western blot analysis was used to confirm the separation by detecting the nuclear marker PARP and the cytoplasmic marker α-tubulin (Figure 2A). Nuclear proteins were enriched with streptavidin by LC–MS/MS, and a total of 289 *M. bovis* proteins were identified (Supplementary Table S2). Among them, the 66 highly abundant proteins with intensity-based absolute quantification (IBAQ) ≥ 0.2% and unique peptides ≥5 were selected as potential nucleomodulins (Table 1). Meanwhile, a ratio between the IBAQ of the 66 proteins identified by the DB approach and the IBAQ of these proteins in the total protein of *M. bovis* (Zhu et al., 2020) was calculated. Eight proteins with a high ratio (ratio > 9) were selected for verification (Figure 2B and Table 1). The 66 potential nucleomodulins of *M. bovis* were further categorized according to COG (Figure 2C), of which 13 proteins were not annotated and 1 protein was not scanned. The most relevant functions of the remaining 52 proteins are “translation, ribosomal structure and biogenesis” (J; 19.2%) and “function unknown” (S; 19.2%). Metabolism-related categories include “energy production and conversion” (C; 13.5%), “amino acid transport and metabolism” (E; 9.6%), “carbohydrate transport and metabolism” (G; 9.6%), “inorganic ion transport and metabolism” (P; 9.6%), “nucleotide transport and metabolism” (5.8%), and “lipid transport and metabolism” (F; 3.8%). Four functional categories were related to “cell wall/membrane/envelope biogenesis” (M; 7.7%), “intracellular trafficking, secretion, and vesicular transport” (U; 1.9%), “transcription” (K; 1.9%), and “replication, recombination, and repair” (L; 1.9%).

**Table 1:** High abundant proteins identified by the DB approach.

Gene ID	Protein name	Type of Secretion	Ratio^#^
Mbov_0016	P48-like surface lipoprotein	Classical	6.03
Mbov_0103	pdhB pyruvate dehydrogenase E1 component subunit beta	No-classical	2.27
Mbov_0579	Membrane lipoprotein P81	Classical	2.68
Mbov_0102	pdhA pyruvate dehydrogenase E1 component subunit alpha	Undefined	0.9
Mbov_0798	Variable surface lipoprotein	Classical	16.06
Mbov_0599	30S ribosomal protein	Undefined	8.44
Mbov_0564	Predicted permeases	No-classical	NA
Mbov_0580	Nuclease	Classical	8.29
Mbov_0106	pdhD dihydrolipoamide dehydrogenase	Undefined	1.31
Mbov_0481	EF-tu elongation factor	Undefined	0.52
Mbov_0570	Putative lipoprotein	Classical	8.88
Mbov_0280	Predicted lipoprotein	Classical	11.25
Mbov_0521	Putative transmembrane protein	No-classical	5.62
Mbov_0312	adh alcohol dehydrogenase	Undefined	0.57
Mbov_0739	Putative lipoprotein	Classical	2.15
Mbov_0275	Putative membrane lipoprotein	Classical	4.72
Mbov_0581	Multiple sugar ABC transporter ATP-binding protein	Undefined	3.61
Mbov_0194	Putative transmembrane protein	Undefined	6.1
Mbov_0299	NADH oxidase	Undefined	1.05
Mbov_0314	30S ribosomal protein	No-classical	4.59
Mbov_0619	50S ribosomal protein	Undefined	3.6
Mbov_0116	Putative lipoprotein	Classical	9.61
Mbov_0582	Multiple sugar ABC transporter permease protein	No-classical	34.51
Mbov_0623	30S ribosomal protein	No-classical	1.42
Mbov_0130	Putative transmembrane protein	No-classical	3.52
Mbov_0274	Putative lipoprotein	Classical	1.91
Mbov_0595	cobalt/nickel ABC transporter ATP-binding protein	No-classical	7.18
Mbov_0062	gapA glyceraldehyde 3-phosphate dehydrogenase	Undefined	0.59
Mbov_0019	Sugar ABC transporter permease	Undefined	4.21
Mbov_0217	Predicted lipoprotein	Classical	1.46
Mbov_0216	Putative transmembrane protein	Undefined	0.91
Mbov_0760	Putative transmembrane protein	Undefined	3.73
Mbov_0440	atpA ATP synthase subunit alpha	No-classical	4.48
Mbov_0594	Cobalt/nickel ABC transporter ATP-binding protein	Undefined	5.29
Mbov_0628	50S ribosomal protein	Undefined	1.97
Mbov_0306	phnD phosphonate ABC transporter Substrate-binding protein	Classical	3.83
Mbov_0675	cpdB 5’nucleotidase	Classical	2.21
Mbov_0129	Putative transmembrane protein	Undefined	4.45
Mbov_0791	ECF transporter S component	Undefined	32.16
Mbov_0699	Putative lipoprotein	Classical	28.86
Mbov_0111	Putative lipoprotein	Classical	0.7
Mbov_0338	adh alcohol dehydrogenase	Undefined	0.92
Mbov_0126	pepP XAA-Pro aminopeptidase	No-classical	1.23
Mbov_0839	lacI family transcriptional regulator	Undefined	3.49
Mbov_0438	atpD ATP synthase subunit beta	No-classical	2.07
Mbov_0326	Predicted secreted acid phosphatase	Classical	3.33
Mbov_0475	Putative lipoprotein	Classical	15.49
Mbov_0571	Hexosephosphate transport protein	Undefined	3.34
Mbov_0593	Cobalt/nickel ABC transporter permease protein	No-classical	7.29
Mbov_0154	Putative transmembrane protein	Undefined	1.28
Mbov_0585	Putative lipoprotein	Classical	1.64
Mbov_0629	50S ribosomal protein	Undefined	0.43
Mbov_0006	Esterase/lipase	Undefined	2.1
Mbov_0131	Phosphoketolase	Classical	0.12
Mbov_0509	atpA ATPase subunit alpha	Undefined	0.91
Mbov_0115	oppF oligopeptide ABC transporter ATP-binding protein	No-classical	0.35
Mbov_0294	rpsB 30S ribosomal protein	No-classical	0.68
Mbov_0612	30S ribosomal protein	No-classical	0.81
Mbov_0100	trxB thioredoxin reductase	No-classical	2.27
Mbov_0099	lgt sialyltransferase	Undefined	0.35
Mbov_0018	Simple sugar ABC transporter ATP-binding protein	Undefined	0.62
Mbov_0089	50S ribosomal protein	No-classical	0.51
Mbov_0723	ulaA ascorbate-specific PTS system enzyme II Acomponent	Undefined	14.06
Mbov_0305	Putative transmembrane protein	Undefined	0.67
Mbov_0056	gpsA glycerol-3-phosphate dehydrogenase	No-classical	2.71
Mbov_0510	Putative transmembrane protein	No-classical	1.32

^#^Protein with Ratio > 9 serve as candidate nucleomodulins for subsequent verification.

### Identification of nucleomodulins by the PBB approach

**Figure 3:**
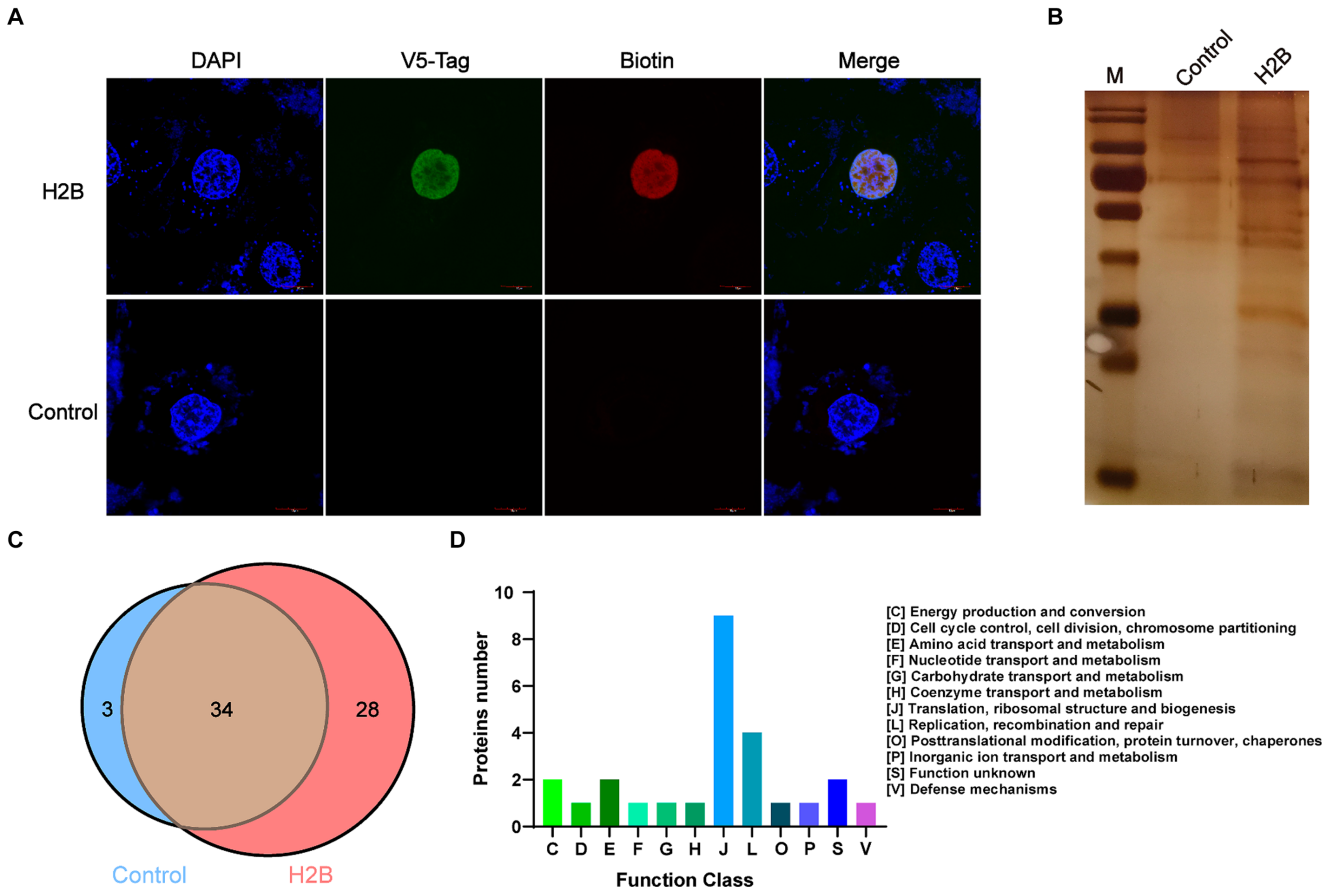
Identification of nucleomodulins by the PBB approach. **(A)** Localization of V5-H2B and biotin observed by confocal microscopy. DAPI: the nuclei labeled with blue; V5-tag: V5-H2B labeled with Green; Biotin: biotin labeled with red. **(B)** Silver staining assay was used to detect the different bands between the control and the H2B groups. **(C)** The venn diagram shows the number of proteins identified in the control and H2B group by LC–MS/MS. **(D)** The COG functional distribution of potential *M. bovis* nucleomodulins by the PBB approach.

As shown in Supplementary Figures S1A,B, IFA and western blotting confirmed that *M. bovis* successfully infected BoMac cells. To screen potential nucleomodulins of *M. bovis* responsible for the infection of BoMac cells, we further used the PBB approach (Figure 1B). When biotin-phenol and H_2_O_2_ were used to treat BoMac cells, V5-H2B-APEX2 (green) and biotin (red) exhibited co-localization within the nucleus, as visualized by confocal microscopy (Figure 3A). The result indicated that H2B-APEX2 was successfully located in the nucleus, and biotin phenols were labeled near H2B-APEX2 after oxidation. Then, the proteins near H2B-APEX2 were enriched by streptavidin from BoMac cell lysis and identified by silver staining (Figure 3B) and LC–MS/MS. The results revealed that 1,375 unique proteins of host cells were identified in BoMac cells expressing V5-H2B-APEX2 (H2B group) (Supplementary Figure S1C and Supplementary Table S3), which are primarily localized in the nucleus (Supplementary Figure S1D and Supplementary Table S4), thus confirming the feasibility of the PBB approach. Furthermore, 62 proteins of *M. bovis* (Supplementary Table S5) were identified in H2B group, while 37 proteins were identified in the control group (Supplementary Table S6). After comparison, 28 unique proteins were identified in the H2B group (Table 2), 3 unique proteins were identified in the control group, and 34 common proteins were identified in both groups (Figure 3C). The 28 potential nucleomodulins were further classified according to COG. Among them, 2 proteins lacked annotations, and functional predictions for the remaining 26 proteins are summarized in Figure 3D. The identified proteins were mostly associated with “translation, ribosomal structure and biogenesis” (J; 34.6%). In addition, the most occupied categories were “replication, recombination and repair” (L; 15.4%), “energy production and conversion” (C; 7.7%), “amino acid transport and metabolism” (E; 7.7%), and “function unknown” (S; 7.7%). There was only one protein in seven functional categories related to “cell cycle control, cell division, chromosome partitioning,” “nucleotide transport and metabolism,” “carbohydrate transport and metabolism,” “coenzyme transport and metabolism,” “posttranslational modification, protein turnover, chaperones,” “inorganic ion transport and metabolism,” and “defense mechanisms.”

**Table 2:** Proteins identified by the PBB approach.

Gene ID	Protein name	Type of Secretion
Mbov_0160	ldhA D-lactate dehydrogenase	Undefined
Mbov_0581	Multiple sugar ABC transporter ATP-binding protein	Undefined
Mbov_0115	oppF oligopeptide ABC transporter ATP-binding protein	Undefined
Mbov_0513	Conserved hypothetical protein	Undefined
Mbov_0599	30S ribosomal protein	Undefined
Mbov_0678	30S ribosomal protein	Undefined
Mbov_0790	Transposase ISMbov1	Undefined
Mbov_0091	Putative transmembrane protein	No-classical
Mbov_0239	Transposase ISMbov4	Undefined
Mbov_0255	CTP synthetase	Undefined
Mbov_0353	adh alcohol dehydrogenase	Undefined
Mbov_0401	Conserved hypothetical protein	Undefined
Mbov_0579	Membrane lipoprotein P81	Classical
Mbov_0663	metK S-adenosylmethionine synthetase	Undefined
Mbov_0049	Putative lipoprotein	Classical
Mbov_0074	pheS phenylalanyl-tRNA synthetase alpha chain	Undefined
Mbov_0127	proS prolyl-tRNA synthetase	Undefined
Mbov_0157	dnaK molecular chaperone DnaK	Undefined
Mbov_0211	Endonuclease I	Classical
Mbov_0228	rnr ribonuclease R	No-classical
Mbov_0334	smc chromosome segregation protein	Undefined
Mbov_0367	Putative transmembrane protein	Undefined
Mbov_0478	gcp O-sialoglycoprotein endopeptidase	Undefined
Mbov_0490	ATP-binding cassette subfamily B	Undefined
Mbov_0533	Ca2 -transporting ATPase	No-classical
Mbov_0660	gltX glutamyl-tRNA synthetase	No-classical
Mbov_0710	rpsP ribosomal protein small subunit S16	Undefined
Mbov_0712	50S ribosomal protein	Undefined

### Verification of the *Mycoplasma bovis* nucleomodulins

**Figure 4:**
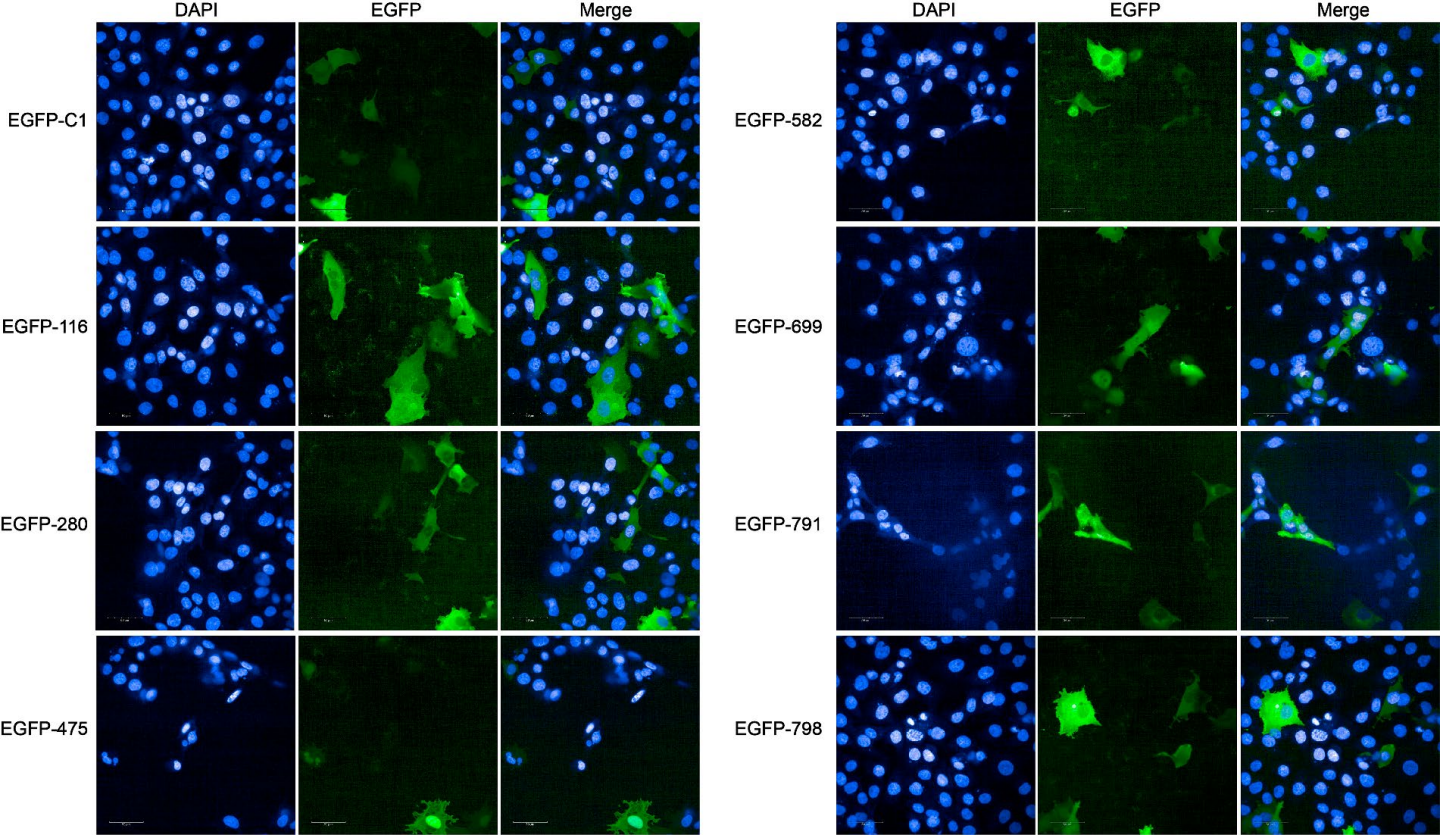
Verification of nucleomodulins by the DB approach. The localization of potential nucleomodulins encoded by pEGFP plasmid (pEGFP-C1, pEGFP-116, pEGFP-280, pEGFP-475, pEGFP-582, pEGFP-699, pEGFP-791, and pEGFP-798) in BoMac was detected by fluorescence microscopy at 24 h post-transfection. The cells transfected with pEGFP-C1 served as control.

In the DB approach, we selected the proteins with the highest enrichment degree of top eight (ratio > 9) as candidate nucleomodulin proteins, including Mbov_0582, Mbov_0791, Mbov_0699, Mbov_0798, Mbov_0475, Mbov_0723, Mbov_0280, and Mbov_0116 (Table 1). We successfully constructed seven corresponding recombinant plasmids expressing EGFP fusion proteins: pEGFP-116, pEGFP-280, pEGFP-475, pEGFP-582, pEGFP-669, pEGFP-791, and pEGFP-798. The BoMac cells transfected with plasmids were observed under the Opera Phenix to detect the cellular location of these EGFP fusion proteins. Figure 4 shows that only EGFP-475 exhibited nuclear localization; the remaining proteins were cytoplasmic.

**Figure 5:**
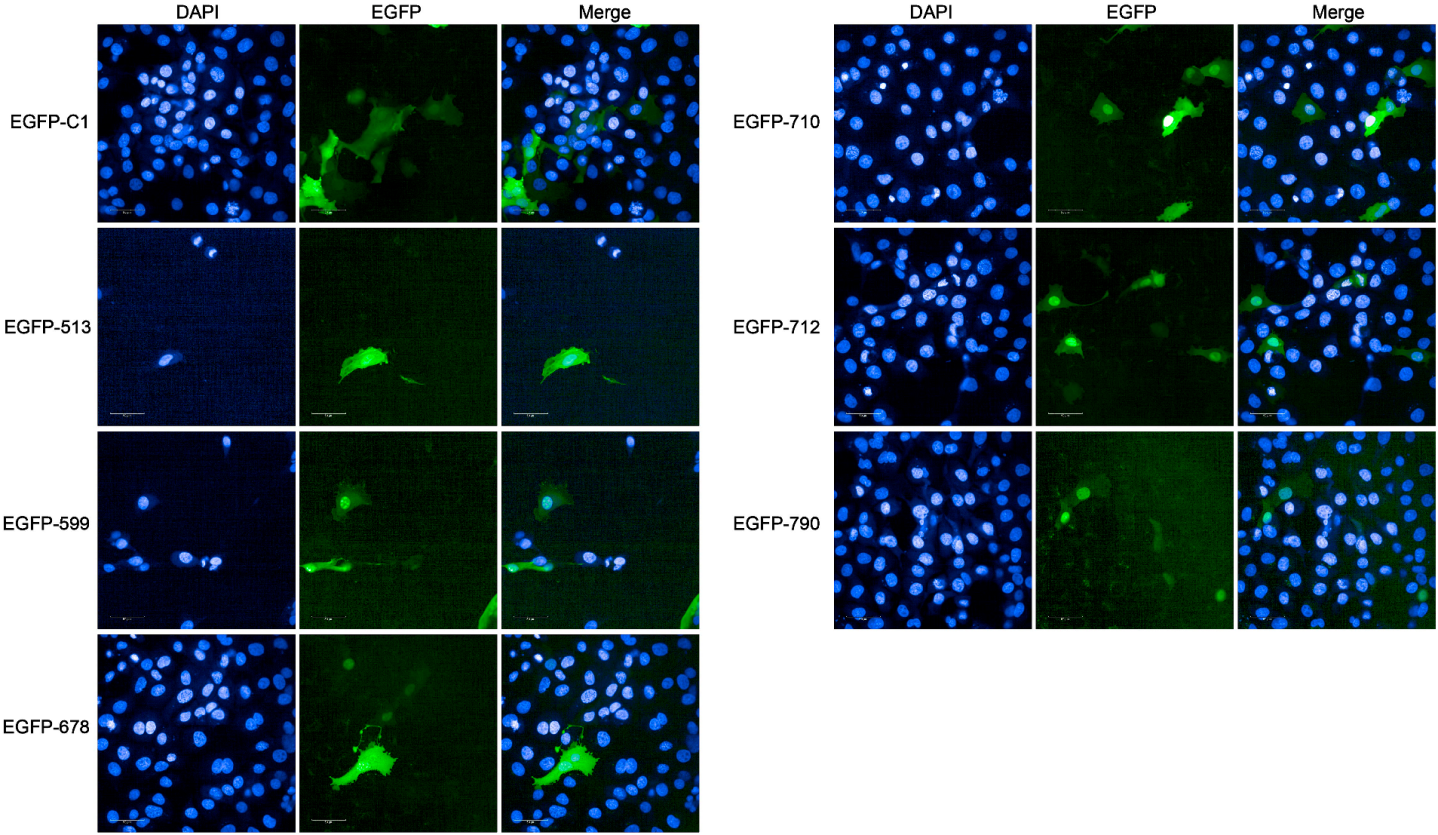
Verification of nucleomodulins by the PBB approach. The localization of potential nucleomodulins encoded by pEGFP plasmids (pEGFP-C1, pEGFP-513, pEGFP-599, pEGFP-678, pEGFP-710, pEGFP-712, and pEGFP-790) in BoMac was detected by fluorescence microscopy at 24 h post-transfection. The cells transfected with pEGFP-C1 served as the control.

In the PBB approach, we excluded four proteins containing transmembrane domains (Mbov_0091, Mbov_0367, Mbov_0490, and Mbov_0533) and successfully constructed 16 corresponding recombinant plasmids. Punctuated green fluorescence showed that six proteins (EGFP-513, EGFP-599, EGFP-678, EGFP-710, EGFP-712, and EGFP-790) were localized in the nuclei (Figure 5), nine proteins (EGFP-049, EGFP-157, EGFP-160, EGFP-255, EGFP-353, EGFP-401, EGFP-478, EGFP-579, and EGFP-663) were mainly located in the cytoplasm after transfection (Supplementary Figure S2), and one protein (EGFP-211) was not expressed.

### Comparative analysis of potential nucleomodulins identified by the DB and PBB approaches

**Figure 6:**
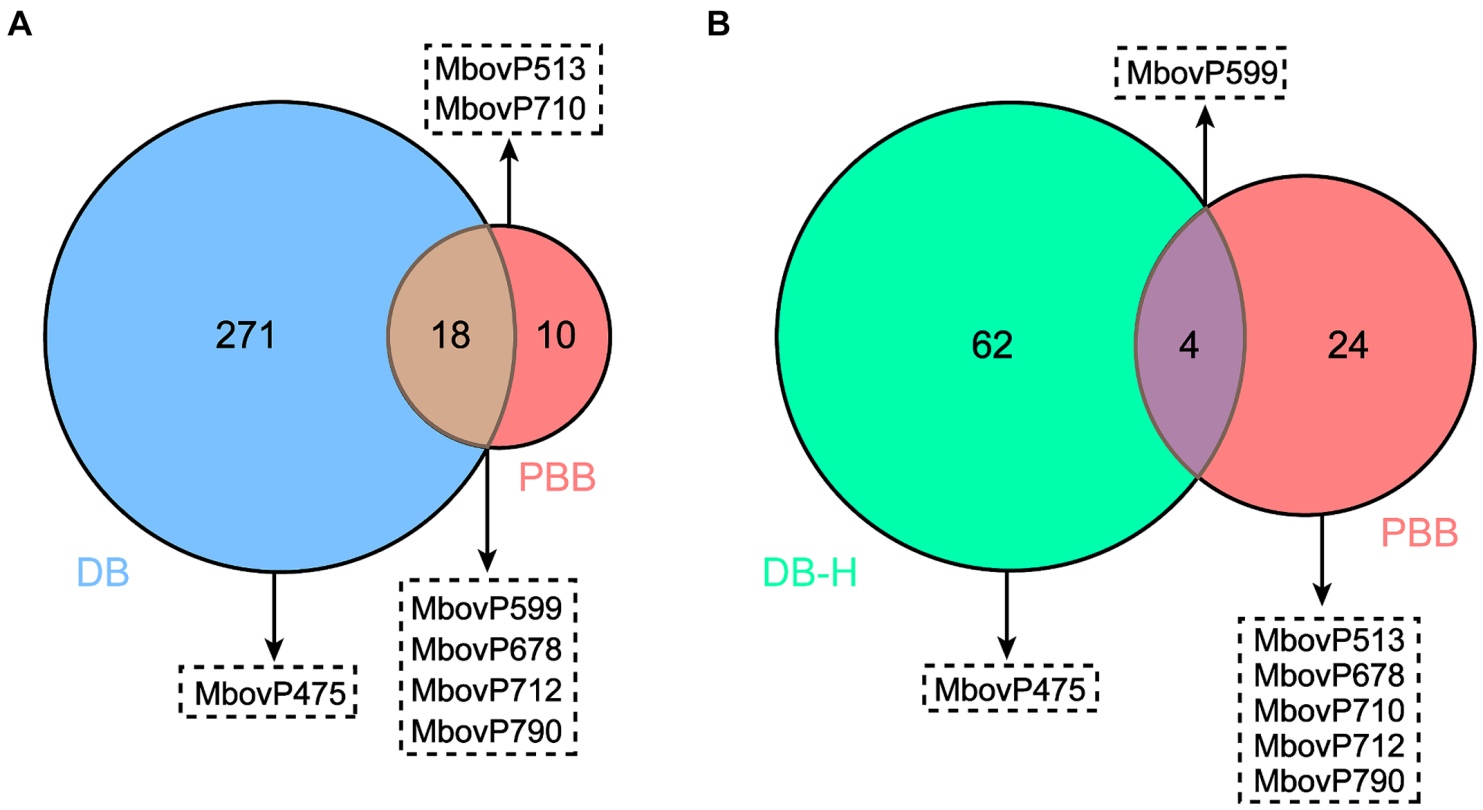
Comparative analysis on potential nucleomodulins identified by the PBB and DB **(A)** or DB-H **(B)** approaches. DB-H: direct biotinylation (high abundance proteins).

We further analyzed the 289 and 28 proteins identified by the DB and PBB approaches, respectively. As shown in Figure 6A, 271 unique proteins were identified by the DB approach, 10 unique proteins were identified by the PBB approach, and 18 proteins overlapped in the two approaches. Among them, the DB approach identified one unique nucleomodulin, the known nuclear located MbovP475; the PBB approach identified two unique nucleomodulins (MbovP513 and MbovP710); and four nucleomodulins (MbovP599, MbovP678, MbovP712, and MbovP790) were identified by both approaches. The overlap of both approaches had the highest positive nucleomodulin identification rate (4/18, 22.2%), followed by the PBB approach (2/10, 20.0%), and finally the DB approach (1/271, 0.4%). In addition, the 66 highly abundant proteins (DB-H) identified by the DB approach were compared with the total 28 proteins identified by the PBB approach. One nucleomodulin was identified from the four overlapped proteins (Figure 6B).

## Discussion

Nucleomodulins are emerging factors that promote pathogen-induced epigenetic modifications and control the host cell epigenome via epigenetics. However, previous studies have focused on the function of a single nucleomodulin. Here, we intended to establish high-throughput approaches to screen nucleomodulins of *M. bovis*. For the DB approach, we initially attempted biotinylated labeling of *M. bovis* secretory proteins for incubation with BoMac cells, but interference from horse serum proteins in PPLO media hindered accurate identification of nucleomodulins. Hence, we swapped to whole protein of *M. bovis*. The results showed that 30% of total *M. bovis* proteins (289/873) was identified. Even though extraction of nuclear proteins from BoMac cells was confirmed to theoretically exclude the contamination of cytoplasmic proteins by western blot assay, it is probable that some proteins of *M. bovis* from the cytoplasm of BoMac exist and were identified by the highly sensitive method LC–MS/MS. Furthermore, the potential nonspecific binding of *M. bovis* proteins to the nuclear membrane of BoMac cells may contribute to false positives. To further remove the interference of cytoplasmic proteins and nonspecific binding to nuclear membrane proteins, we excluded proteins with low abundance and nonspecific peptides (intensity-based absolute quantification IBAQ<0.2% and unique peptides <5). Finally, 66 proteins were selected and compared with the total protein abundance of *M. bovis* to calculate the enrichment ratio, and seven highly enriched proteins were selected for verification. However, the reported nucleomodulin MbovP475 was the only protein verified to enter BoMac nuclei for this technique. Subsequently, we used the PBB approach to screen the nucleomodulins of *M. bovis* during infection. APEX2 can precisely target and label proteins within a 20 nm radius in live cells. LC–MS/MS analysis of surrounding proteins of APEX-generated biotinylated proteins not only revealed various unknown proteins in diverse cellular compartments (Lee et al., 2016), but was also used to study host-pathogen interaction in *Chlamydia trachomatis* (Rucks et al., 2017; Dickinson et al., 2019; Olson et al., 2019). Using the PBB approach, we confirmed that the APEX2-H2B fusion proteins were located in BoMac nuclei, and identified a total of 28 unique proteins of *M. bovis*. Finally, we verified six proteins located in the BoMac nuclei. COG analysis highlighted “translation, ribosomal structure, and biogenesis” as the primary protein enrichment identified by PBB and DB approaches, aligning with our experimental findings that ribosomes efficiently enter the BoMac nucleus. Compared with DB, The PBB approach captures proteins proximal to nuclei during natural *M. bovis* infection of BoMac cells, providing realistic results. However, the enrichment of numerous host nuclear proteins during proximity labeling hinders the detection of trace nucleomodulins, resulting in fewer candidates for further screening. Meanwhile, this was likely mainly due to the low transfection efficiency of BoMac cells. Therefore, it was possible to increase the number of selected proteins by constructing cell lines that stably expressed H2B-APEX2. For the PBB approach, our substantial addition of *M. bovis* protein during *in vitro* incubation with BoMac cells elevated the probability of either subdued *M. bovis* expression or limited nuclear translocation, thereby enhancing the detection of trace nucleomodulins signals and providing more potential nucleomodulins than the DB approach. However, the half-life of the biotin-labeled *M. bovis* proteins and the efficiency of biotin-labeled differential *M. bovis* proteins could decrease the ability of the DB approach to identify the nucleomodulins. Moreover, combining the DB and PBB approaches enhanced the positive rate of nucleomodulins screening. Generally, successful separation of nuclear and cytoplasmic proteins is important to identify nucleomodulins by the DB approach. The expression of APEX2 in the subcellular location of each cell is important to identify nucleomodulins by the PBB approach; the proteins overlapping between the two approaches could increase our ability to identify nucleomodulins. In addition, in this study, we used GFP fusion proteins to map protein nuclear localization but realized that overexpression may not accurately reflect endogenous expression during *M. bovis* infection. In the future, developing antibodies, performing transcriptional profiling, or using inducible promoters for ectopic expression will provide more accurate insight into protein spatial expression, secretion and localization.

Nucleomodulin is a class of secreted proteins of pathogenic bacteria. However, according to secretory signal peptide prediction, only the nucleomodulin MbovP475 has a classical LIPO signal peptide. The other six proteins were undefined (Table 3). Among these undefined proteins, there are four ribosomal related proteins, one transposable protein, and one conserved hypothetical protein. Our previous data revealed that ribosomal proteins (MbovP599, MbovP678, MbovP710, and MbovP712) and transposase (MbovP790) were presented in the secreted proteome of *M. bovis* (Zubair et al., 2020; Zhang et al., 2021). In accordance with our data, a large number of ribosomal proteins were also identified in the extracellular vesicles of *M. agalactiae* 5,632, *M. mycodies* subsp. *mycoides* Afadé, and *Acholeplasma laidlawii* PG8 (Chernov et al., 2014; Gaurivaud et al., 2018). Consistent with our findings, the DNA methyltransferases Mhy1, Mhy2, and Mhy3 of *M. hyorhinis* (Chernov et al., 2015) entered the cell nuclei without a signal peptide. Previous *in vivo* studies showed the occasional intracellular localization of *M. bovis* within inflammatory host cells (Rodriguez et al., 1996; Kleinschmidt et al., 2013). *M. bovis* is also capable of invading primary embryonic calf turbinate (PECT) cells, embryonic bovine lung (EBL) cells, and embryonic bovine tracheal (EBTr) cells (Bürki et al., 2015; Suleman et al., 2016). Therefore, these proteins without signal peptides may be secreted through extracellular vesicles or released from lysed mycoplasma in host cells, and then enter the host cell nuclei.

**Table 3:** Validation of the potential nucleomodulins.

Gene ID	Protein name	Protein family membership	Type of Secretion	Approach
Mbov_0475	Putative lipoprotein	Protein of unknown function DUF 285	Classical	DB
Mbov_0513	Conserved hypothetical protein	Protein of unknown function DUF2714	Undefined	PBB
Mbov_0599	30S ribosomal protein	Rbiosomal_S13 superfamily rpsM	Undefined	DB&PBB
Mbov_0678	30S ribosomal protein	Ribosomal_S12_like superfamily rps12	Undefined	DB&PBB
Mbov_0710	rpsP ribosomal protein small subunit S16	RpsP superfamily RpsP	Undefined	PBB
Mbov_0712	50S ribosomal protein	Ribosomal_L19 superfamily rp1S	Undefined	DB&PBB
Mbov_0790	Transposase ISMbov1	Transpos_IS30	Undefined	DB&PBB

Nucleomodulin is an emerging family of bacterial effectors that enter the nuclei to regulate gene expression and thereby regulate eukaryotic cellular processes (Bierne and Pourpre, 2020; Hanford et al., 2021). The functions of nucleomodulins include activating host transcription, altering host nuclear homeostasis and modifying host DNA and histones (Hanford et al., 2021). For example, the Ptpa of *M. tb* enhances asymmetric dimethylation of histone H3 arginine 2 (H3R2me2a) by targeting protein arginine methyltransferase 6 (PRMT6), inhibiting glutathione peroxidase 4 (GPX4) expression and eventually inducing ferroptosis to promote *M. tb* pathogenicity and dissemination (Qiang et al., 2023). *Brucella abortus* nucleomodulin BspJ may directly or indirectly regulate macrophage apoptosis through interaction with *Homo sapiens* NME/NM23 nucleoside diphosphate kinase 2 (NME2) and *Homo sapiens* rib creatine kinase B (CKB) to promote the survival of intracellular *Brucella* (Ma et al., 2020). In our study, seven proteins were verified as nucleomodulins. MbovP475 is involved in the regulation of host gene transcription (Zhao et al., 2022). The other four ribosomal related-proteins, one transposable protein, and one conserved hypothetical protein, were novel nucleomodulins revealed by this study. The ribosome synthesizes proteins under the direction of mRNA in both prokaryotic and eukaryotic cells. Although the composition of ribosomes can differ, their basic structures are highly conserved in all cellular organisms (Ban et al., 2014). Notably, *Spiroplasma citri*, a bacterium belonging to the class Mollicutes, has been characterized for its ribosomal protein’s DNA binding capacity (Le Dantec et al., 1998). A transposon is a piece of DNA that can change its position by moving or jumping freely in the genome, using its the efficient insertion ability to create mutation (Zhu et al., 2020). It also has the ability to effectively carry exogenous genes and integrate them into the target genome (Kahlig et al., 2010). Ribosomal proteins and transposases play an important role in the regulation of prokaryotic and eukaryotic organisms. However, whether these proteins enter the host cell nuclei to regulate host genes remains to be determined.

## Conclusion

Our study provided the DB and PBB approaches to screen potential nucleomodulins of *M. bovis*. A total of seven nucleomodulins candidates were confirmed to enter the BoMac nuclei including one known (MbovP475) nucleomodulins and six novel ones. Therefore, both techniques are very efficient to identify potential nucleomodulins.

## Data availability statement

The LC-MS/MS data in this study were deposited in the iProx via the project ID: IPX0008669000. The LC-MS/MS data also have been deposited to the ProteomeXchange Consortium (https://proteomecentral.proteomexchange.org) via the iProX partner repository with the dataset identifier PXD051637.

## Author contributions

DL: Conceptualization, Data curation, Formal analysis, Methodology, Software, Validation, Visualization, Writing – original draft, Writing – review & editing. JioC: Data curation, Formal analysis, Validation, Writing – review & editing. MZ: Data curation, Formal analysis, Validation, Writing – review & editing. YF: Data curation, Formal analysis, Validation, Writing – review & editing. AR: Formal analysis, Writing – review & editing. YC: Data curation, Formal analysis, Writing – review & editing. XC: Data curation, Formal analysis, Writing – review & editing. CH: Data curation, Formal analysis, Writing – review & editing. JiaC: Data curation, Formal analysis, Writing – review & editing. ES: Data curation, Formal analysis, Writing – review & editing. GZ: Conceptualization, Data curation, Formal analysis, Methodology, Software, Validation, Visualization, Writing – review & editing. AG: Conceptualization, Data curation, Formal analysis, Funding acquisition, Resources, Supervision, Validation, Writing – review & editing, Project administration.

## Footnotes

^1^ https://services.healthtech.dtu.dk/services/SignalP-6.0/

^2^ http://www.cbs.dtu.dk/services/SecretomeP

^3^ http://eggnog5.embl.de/#/app/seqscan

^4^ www.UniProt.org
