# Supplementary figures and images for: Identification of potential nucleomodulins of Mycoplasma bovis by direct biotinylation and proximity-based biotinylation approaches
Source: Front Microbiol. 2024 Jul 9;15:1421585. doi: 10.3389/fmicb.2024.1421585 (PMC11263210; doi:10.3389/fmicb.2024.1421585)

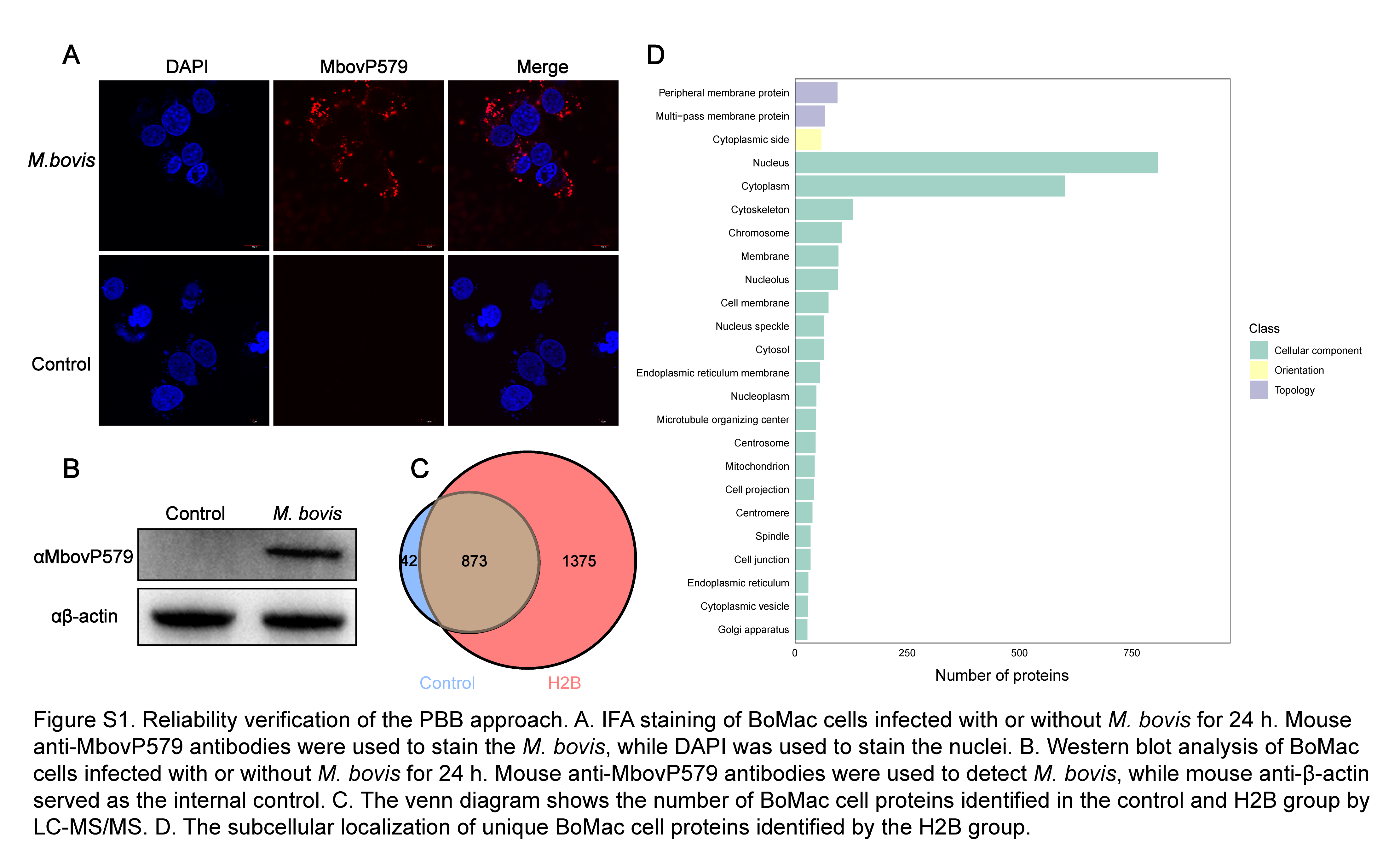

Supplement: Supplementary file 1 [file Image_1.tif]

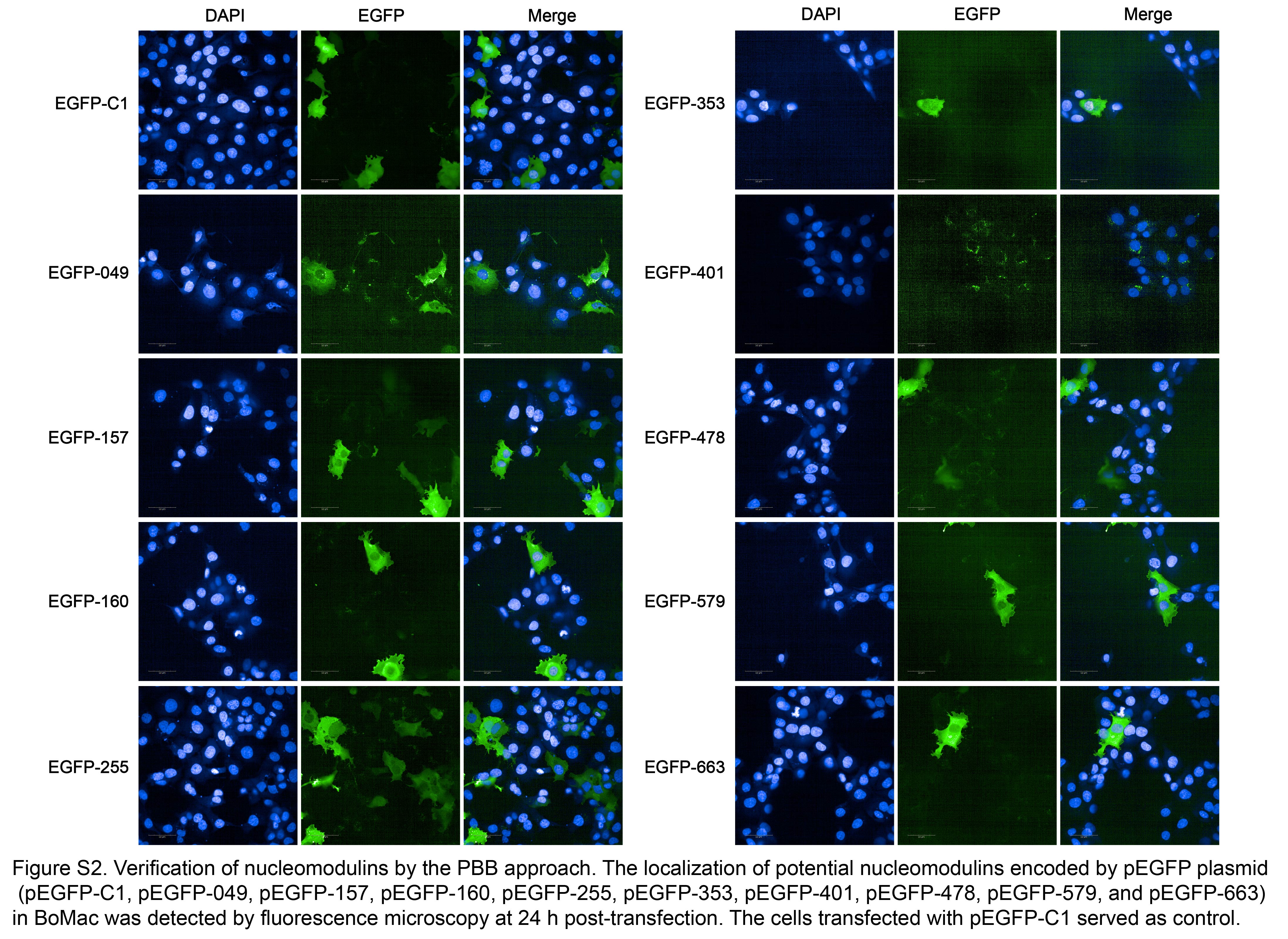

Supplement: Supplementary file 2 [file Image_2.jpg]
